# Low number of neurosecretory vesicles in neuroblastoma impairs massive catecholamine release and prevents hypertension

**DOI:** 10.3389/fendo.2022.1027856

**Published:** 2022-12-01

**Authors:** Annick Mühlethaler-Mottet, Silvia Uccella, Deborah Marchiori, Stefano La Rosa, Jean Daraspe, Katia Balmas Bourloud, Maja Beck Popovic, Philippe J. Eugster, Eric Grouzmann, Karim Abid

**Affiliations:** ^1^ Pediatric Hematology-Oncology Research Laboratory, Woman-Mother-Child Department, Lausanne University Hospital and University of Lausanne, Lausanne, Switzerland; ^2^ Department of Biomedical Sciences, Humanitas University, Milan, Italy; ^3^ Pathology Service, Istituti di Ricovero e Cura a Carattere Scientifico (IRCCS) Humanitas Research Hospital, Milan, Italy; ^4^ Unit of Pathology, Department of Medicine and Surgery, University of Insubria, Varese, Italy; ^5^ Institute of Pathology, Department of Laboratory Medicine and Pathology, University of Lausanne, Lausanne, Switzerland; ^6^ Electron Microscopy Facility (EMF), University of Lausanne, Lausanne, Switzerland; ^7^ Pediatric Hematology Oncology Unit, Woman-Mother-Child Department, Lausanne University Hospital and University of Lausanne, Lausanne, Switzerland; ^8^ Service of Clinical Pharmacology and Toxicology, Lausanne University Hospital, Lausanne, Switzerland

**Keywords:** neuroblastoma, pheochromocytoma, catecholamine, metanephrine, biomarkers, neurosecretory vesicles, chromaffin cell differentiation

## Abstract

**Introduction:**

Neuroblastoma (NB) is a pediatric cancer of the developing sympathetic nervous system. It produces and releases metanephrines, which are used as biomarkers for diagnosis in plasma and urine. However, plasma catecholamine concentrations remain generally normal in children with NB. Thus, unlike pheochromocytoma and paraganglioma (PHEO/PGL), two other non-epithelial neuroendocrine tumors, hypertension is not part of the usual clinical picture of patients with NB. This suggests that the mode of production and secretion of catecholamines and metanephrines in NB is different from that in PHEO/PGL, but little is known about these discrepancies. Here we aim to provide a detailed comparison of the biosynthesis, metabolism and storage of catecholamines and metanephrines between patients with NB and PHEO.

**Method:**

Catecholamines and metanephrines were quantified in NB and PHEO/PGL patients from plasma and tumor tissues by ultra-high pressure liquid chromatography tandem mass spectrometry. Electron microscopy was used to quantify neurosecretory vesicles within cells derived from PHEO tumor biopsies, NB-PDX and NB cell lines. Chromaffin markers were detected by qPCR, IHC and/or immunoblotting.

**Results:**

Plasma levels of metanephrines were comparable between NB and PHEO patients, while catecholamines were 3.5-fold lower in NB vs PHEO affected individuals. However, we observed that intratumoral concentrations of metanephrines and catecholamines measured in NB were several orders of magnitude lower than in PHEO. Cellular and molecular analyses revealed that NB cell lines, primary cells dissociated from human tumor biopsies as well as cells from patient-derived xenograft tumors (NB-PDX) stored a very low amount of intracellular catecholamines, and contained only rare neurosecretory vesicles relative to PHEO cells. In addition, primary NB expressed reduced levels of numerous chromaffin markers, as compared to PHEO/PGL, except catechol O-methyltransferase and monoamine oxidase A. Furthermore, functional assays through induction of chromaffin differentiation of the IMR32 NB cell line with Bt2cAMP led to an increase of neurosecretory vesicles able to secrete catecholamines after KCl or nicotine stimulation.

**Conclusion:**

The low amount of neurosecretory vesicles in NB cytoplasm prevents catecholamine storage and lead to their rapid transformation by catechol O-methyltransferase into metanephrines that diffuse in blood. Hence, in contrast to PHEO/PGL, catecholamines are not secreted massively in the blood, which explains why systemic hypertension is not observed in most patients with NB.

## Introduction

Neuroendocrine neoplasms encompass a large diversity of epithelial and non-epithelial neoplasms differing for their incidence, localization, morphology, biology and available treatments. Despite these facts, these tumors share common characteristics, including the potential ability of hormones and biogenic amine production and secretion (1–4). Pheochromocytoma (PHEO), paraganglioma (PGL) and neuroblastoma (NB) are non-epithelial neuroendocrine neoplasms arising from the adrenal medulla and ganglia of the sympathetic nervous system (SNS), which typically produce and secrete catecholamines (CATs; dopamine, DA, norepinephrine NE, epinephrine, E) (5). However, whereas PHEO and PGL typically occur in adults and are composed of differentiated chromaffin cells or sympathetic neurons, respectively, NB is a pediatric neoplasm resulting from the abnormal differentiation and maturation of sympathetic progenitors derived from the embryonic trunk neural crest (6). NB is the second most common solid tumor in children, and can occur anywhere in the sympathetic chain, but most commonly in the abdominal region and adrenal medulla. Fifty percent of NB cases are diagnosed before the age of 2 years and 90% before the age of 5 years. Hypertension, a typical clinical symptom of CAT-producing tumors, has a low prevalence in NB, ranging from 2.2% to 10% in two recent reports and is mainly related to renal artery compression, which frequently resolves after tumor resection (7, 8).

CAT metabolism has been reviewed extensively elsewhere (2, 3). Briefly, CAT production occurs primarily in adrenal chromaffin cells and sympathetic nerves. It starts in the cytoplasm from L-tyrosine, which is converted to dihydroxyphenylalanine (DOPA) by the rate-limiting enzyme tyrosine hydroxylase (TH), and DOPA is further converted to DA by L-aromatic amino acid decarboxylase (DDC). In chromaffin cells, DA is internalized by vesicular monoamine transporters (SLC18A1 and SLC18A2, formerly VMAT1 and VMAT2, respectively) into neurosecretory vesicles where it is converted to NE by dopamine beta-hydroxylase (DBH). Subsequently, chromaffin adrenergic cells expressing the enzyme phenylethanolamine-N-methyltransferase (PNMT) synthesize E from NE. Following sympathetic stimulation, CATs stored in neurosecretory vesicles are secreted into the bloodstream to reach their receptors and trigger the described “fight or flight response” (9). A small proportion of the CATs that leak from the neuroendocrine vesicles is converted in the cytoplasm to metanephrines (MNs) by the COMT (catechol O-methyltransferase) enzyme. MNs refers to the three metabolites methoxytyramine (MT), normetanephrine (NMN), and metanephrine (MN), generated from DA, NE and E, respectively. NE and DA in the cytoplasm can also undergo oxidation to dihydroxyphenol glycol (DHPG) and 3,4-dihydroxyphenylacetic acid (DOPAC), respectively, by monoamine oxidase A (MAOA). MNs, DHPG and DOPAC diffuse freely across the membrane and are released into the bloodstream (10, 11).

Few reports have been published on the mechanisms responsible for CATs synthesis and release in NB cells compared with normal chromaffin cells. The majority of studies have focused on PHEO/PGL and it has been shown that the excess concentration of CATs found in these tumors and in the plasma of the affected patient was a consequence of the overexpression of TH and DBH enzymes involved in CATs synthesis (12, 13). NB were shown to produce CATs, especially, DA and NE (14, 15) but no E due to the lack of expression of PNMT in NB (16, 17). In contrast to PHEO/PGL, NB contain few storage/neurosecretory vesicles and low amount of CATs in tumor tissues (18). Nevertheless, NMN and especially MT, which arise from NE and DA, respectively, represent potent biomarkers of NB in plasma and urine (19). In this study, we explore the metabolism of CATs in NB versus PHEO/PGL and decipher at the molecular level the mechanism that prevents NB from inducing hypertension, an expected clinical sign for a CAT-producing neoplasm.

## Material and methods

### NB and PHEO/PGL tissues and plasma collection

PHEO/PGL tissue samples were carefully selected by the surgeon or pathologist to be free of remaining healthy adrenal tissue. NB tumor material was collected from patients with high-risk L2 and M stage NB diagnosed at the Hemato-Oncology Unit of the University Hospital of Lausanne, Switzerland, enrolled in the European International Collaboration for Neuroblastoma Research (SIOPEN) HR-NBL1 study, after informed consent and in accordance with local institutional ethical regulations. For plasma collection, samples were collected through a forearm venous cannula with the patient held in a supine position for at least 15 minutes before collection. Patients’ relatives or nurses were informed of the need to fast 24 hours before blood collection, when possible. All samples were collected on ice and centrifuged within 30 minutes of puncture at 2500g for 10 minutes at 4°C. Plasma was stored at -80°C until analysis. The NB patient cohort for plasma collection consists of cases at diagnosis representing of all stages, ages, MYCN amplified and non-amplified cases. Quantification of MNs and CATs in plasma was performed as part of the NB or PHEO/PGL diagnostic exclusion test. The complete list of patient samples used for the various analyses is reported in **Supplementary Tables 1A, B**. The available clinical data for the PHEO patients included in our study are reported in **Supplementary Table 2**, with the newly proposed three cluster classification (20). This study was approved by the local ethics committee of the canton of Vaud (reference numbers: 2017-01865, 95/04 and 26/05).

### NB xenograft

All *in vivo* procedures were performed in accordance with the guidelines of the Swiss Ordinance on Animal Protection and the Ordinance on Animal Experiments of the Federal Veterinary Office (FVO). The animal testing protocols were approved by the Swiss FVO (authorization number: VD2995). All reasonable efforts were made to reduce suffering, including anesthesia for painful procedures.

The NB-PDX material used in this study was derived from a previous study (17), except for the NB12-BM-2 model. The latter NB-PDX was generated from NB cells isolated from bone marrow aspirate of a patient at the time of diagnosis (male, 18 months at diagnosis, stage 4, MYCN amplified) and maintained *in vitro* for a limited number of passages in neuronal basal medium (<5). Primary NB cells (1*10^6^) were suspended in 200 µl of Dulbecco Modified Eagle DMEM medium (Invitrogen, Luzern, Switzerland) and BD Matrigel Basement Membrane matrix (1:1; BD Biosciences, San Diego, CA, USA) and implanted subcutaneously (s.c.) into the flanks of athymic Swiss nude mice (Charles River Laboratories, France). Tumor growth was monitored using calipers every 3 days. Mice were sacrificed when tumors reached a volume of approximately 900 mm3. NB12-BM-2 correspond to the second *in vivo* passage of subcutaneous transplants. Tumor fragments were divided into pieces for paraffin-embedded tissue formation, or collected in 0.1 M perchloric acid for quantification of CATs and MNs, or snap-frozen in liquid nitrogen for protein or RNA extraction. NB xenograft fragments were also dissociated using the Mouse Tumor Dissociation Kit (Miltenyi Biotec GmbH, Germany) according to the manufacturer’s instructions and filtered through CellTricks (50 μm; Partek, Inc, St Louis, MO, USA).

### RNA extraction and real-time qPCR

RNA extraction was performed from fragmented tumor tissue with a micropotter using Trizol (Invitrogen, Luzern, Switzerland) and for cell lines using the RNAeasy kit with DNaseI treatment according to the manufacturer’s instructions (Qiagen, Hombrechtikon, Switzerland). The synthesis of cDNA was performed from 1 µg of RNA using the PrimeScriptTM RT reagent kit (Takara Bio Inc, Japan). Real-time qPCR analyses for tumor tissues were performed in 384 wells using Sybergreen (Roche, Basel, Switzerland) as follows: 10 min at 95°C, 40 cycles of 15 sec at 95°C, 1 min at 60°C with the Applied Biosystems 7900HT SDS (Thermo Fischer Scientific, Reinach, Switzerland). Normalization of gene expression was performed on the three reference genes (RG) TBP, EEIF1A1, and GAPDH using the ΔCt method with Ct_RG = (Ct_ TBP+Ct_GAPDH+Ct_EEIF1A1)/3 and mRNA expression ratio = 2^-(Ct_GeneX-Ct_RG)^.

Real-time qPCR for *in vitro* assay were performed in duplicates using the QuantiFast SYBR^®^ green reagent (Qiagen, Hilden, Germany). Cycling conditions were: 5 min at 95°C, 40 cycles of 10 sec at 95°C, 30 sec at 60°C, and 1 sec at 72°C with the Rotor Gene 6000 real-time cycler (Corbett, Qiagen). Normalization of gene expression was performed on the two reference genes (RG) HPRT1 and SDHA using the ΔCt method with Ct_RG = (Ct_ HPRT1+Ct_SDHA)/2 and mRNA expression ratio = 2^-(Ct_GeneX-Ct_RG)^.

Primers were chosen with the primer designing tool from the National Center for Biotechnology Information (NCBI) and described in the **Supplementary Table 3**.

### Immunoblotting

Tumor tissues were disaggregated and lyzed with a micropotter in lysis buffer (1x PBS, 0.5% triton X-100, and 1x protease inhibitor cocktail (Complete mini, EDTA-free, Roche, Mannheim, Germany) to represent 20% w/v. The tissue lysates were then sonicated and centrifuged at 2000 g for 30 seconds to remove pellets. Samples were fractionated by SDS-PAGE under reducing conditions using precast gels (Bio-Rad, Reinach, Switzerland). The loaded volumes were 10 µl from a 20% w/v extract. Next, proteins were transferred to a nitrocellulose membrane (Amersham Milian, Geneva, Switzerland) and probed with primary antibodies against SYP (1/1000, Cusabio Tech, ref. CSB-PA0004215 Chemie Brunschwig, Basel, Switzerland), TH (1/1000, Millipore, Zug, Switzerland ref AB-152), DBH (1/5000, generated against full-length recombinant human DBH protein (21) and ACTB (AC-15, 1/5000 Sigma-Aldrich Chemie, Buchs, Switzerland). HRP-conjugated anti-mouse and anti-rabbit secondary antibodies were from Bio-Rad (Cat. Nos. 170-6516 and 170-6515, respectively and diluted 4000x). Immunoreactive bands were revealed by a chemiluminescence assay (PerkinElmer, Schwerzenbach, Switzerland) and the signal was processed by a digital image analyser (ImageQuant LAS-4000, General Electric, Glattbrugg, Switzerland) and quantified using ImageJ software.

### Tumor dissociation and primary cell culture

Tumor tissues were cut into small pieces and digested with collagenase (1mg/ml) (Sigma) in Dulbecco’s modified Eagle’s medium (DMEM, Invitrogen), with shaking at 37°C until complete dissolution of tumor pieces. Cells were washed three times by centrifugation (235g for 2 minutes) and suspended in DMEM supplemented with 10% fetal bovine serum (Invitrogen), 100 U/ml penicillin G, and 100μg/ml streptomycin sulfate (Sigma) and seeded into 24-well plates. After 48 h of incubation in a humidified incubator at 37°C and 5% CO_2_, the cell medium was collected, the cells were washed in PBS and lysed in 100μl of lysis buffer (0.1% tween 20) before quantification of CATs and MNs. Normalization of CATs and MNs quantification to protein levels was performed using a BCA assay (Thermofischer, Reinach, Switzerland) according to the manufacturer’s protocol.

### CAT and MN quantification

Tumor tissues and cells (primary and from cell lines) were disaggregated in lysis buffer (0.1% tween 20) and sonicated using a Branson Sonifier 450 (Branson, Danbury, CT, USA) at full power for 30 seconds. CATs in plasma (free forms) and MNs in tissue and cultured cells (free forms) and MNs in plasma (total forms, which consist of free and SO4-conjugated forms) were extracted using activated alumina (for CATs) or solid-phase extraction (for MNs) and quantified by ultra-high pressure liquid chromatography tandem mass spectrometry (UHPLC-MS/MS) (22–24).

### Cellular differentiation and exocytosis

The established human NB cell lines (SH-SY5Y and IGR-NB8) were obtained from their home laboratory and IMR32 from ATCC. Authentication of the SH-SY5Y and IMR32 cell lines used for the functional assay was performed by microsatellite short tandem repeat analysis before starting the transduction experiments (Microsynth, Switzerland). SH-SY5Y and IMR32 cell lines were incubated for 4 days with Bt2cAMP (Dibutyryl cAMP, N6,2′-O-Dibutyryladenosine 3′,5′-cyclic monophosphate sodium from Sigma) at 500nM in DMEM supplemented with 10% fetal bovine serum (Invitrogen), 100 U/ml penicillin G, and 100μg/ml streptomycin sulfate (Sigma) in 6-well plates in a humidified 5% CO_2_ incubator at 37°C. Cell medium was collected and cells were resuspended in cold PBS, washed in PBS, and lyzed in 100μl of 0.1% TX-100 before quantification of CATs and MNs or used for RNA extraction as described above. For exocytosis experiments, the cell medium was removed and incubated with pre-warmed Krebs buffer (25) containing 56mM KCl or 100 µM nicotine for 45 minutes. After incubation, the cell medium was collected and the cells were resuspended and lyzed before quantification of CATs and MNs.

### Electron microscopy and vesicle counting

Cells were fixed in glutaraldehyde solution (EMS, Hatfield, PA, USA) 2.5% in Phosphate Buffer (PB 0.1M pH7.4) (Sigma, St Louis, MO, USA) during 1 hour at room temperature (RT). Then they were directly postfixed by a fresh mixture of osmium tetroxide 1% (EMS, Hatfield, PA, US) with 1.5% of potassium ferrocyanide (Sigma, St Louis, MO, US) in PB buffer during 1 hours at RT. The samples were then washed three times in distilled water and spin down in low melting agarose 2% in H_2_O (Sigma, St Louis, MO, US), let to solidify on ice, cut in 1mm^3^ cube and dehydrated in acetone solution (Sigma, St Louis, MO, US) at graded concentrations (30%-40min; 50%-40min; 70%-40min; 100%-2x1h). This was followed by infiltration in Epon (Sigma, St Louis, MO, US) at graded concentrations (Epon 1/3 acetone-2h; Epon 3/1 acetone-2h, Epon 1/1-4h; Epon 1/1-12h) and finally polymerized for 48h at 60°C in oven. Ultrathin sections of 50nm were cut on a Leica Ultracut (Leica Mikrosysteme GmbH, Vienna, Austria) and picked up on a copper slot grid 2x1mm (EMS, Hatfield, PA, US) coated with a polystyrene film (Sigma, St Louis, MO, US). Sections were poststained with uranyl acetate (Sigma, St Louis, MO, US) 2% in H_2_O during 10 minutes, rinsed several times with H_2_O followed by Reynolds lead citrate in H_2_O (Sigma, St Louis, MO, US) during 10 minutes and rinsed several times with H_2_O. Two montages (8x8 tiles) per conditions with a pixel size of 9.48nm over an area of 120x120µm were taken with a transmission electron microscope Philips CM100 (Thermo Fisher Scientific, Waltham, MA USA) at an acceleration voltage of 80kV with a TVIPS TemCam-F416 digital camera (TVIPS GmbH, Gauting, Germany). The stereology analysis was performed using 3Dmod and its stereology plugin (IMOD software) (26). Briefly, a grid (500nm spacing) was applied on each montage and each intersection was defined as being part of the vesicles, nucleus and cytoplasm, allowing to determine the percentage of vesicles volume per cell volume.

### Immunohistochemistry

Immunohistochemical analyses were performed on 3 µm thick microtomal sections obtained from formalin-fixed, paraffin-embedded tissue samples using specific commercially available antibodies directed against Chromogranin A, (CHGA) (mouse monoclonal, clone LK2H10, Ventana, Roche Diagnostic Corporation, Indianapolis, IN, USA), Synaptophysin (SYP) (rabbit monoclonal, clone SP11, Ventana), Tyrosine Hydroxylase (TH) (mouse monoclonal, clone 1B5, Novocastra, Newcastle, UK) and SLC18A2 (rabbit polyclonal, Chemicon, Temecula, CA, USA). Immunostains were performed manually as previously described (27), slides were observed under a light microscope (DM2000, Leica Microsystems, Wetzlar, Germany), and the presence of a positive immunoreaction was visualised as cytoplasmic brown stain. The results were scored semi-quantitatively as the percentage of positive cells in relation to the total, as well as with respect to intensity (+, weak; ++, moderate; +++, strong).

### Statistics

The measurement data were explored statistically and graphically using Prism (v. 9.1.0, GraphPad Software, Inc. La Jolla, CA, USA). Methods used are described in the figure legends.

## Result

### NB tissues display massively reduced concentrations of MNs and CATs compared to PHEO/PGL

**Figure 1:**
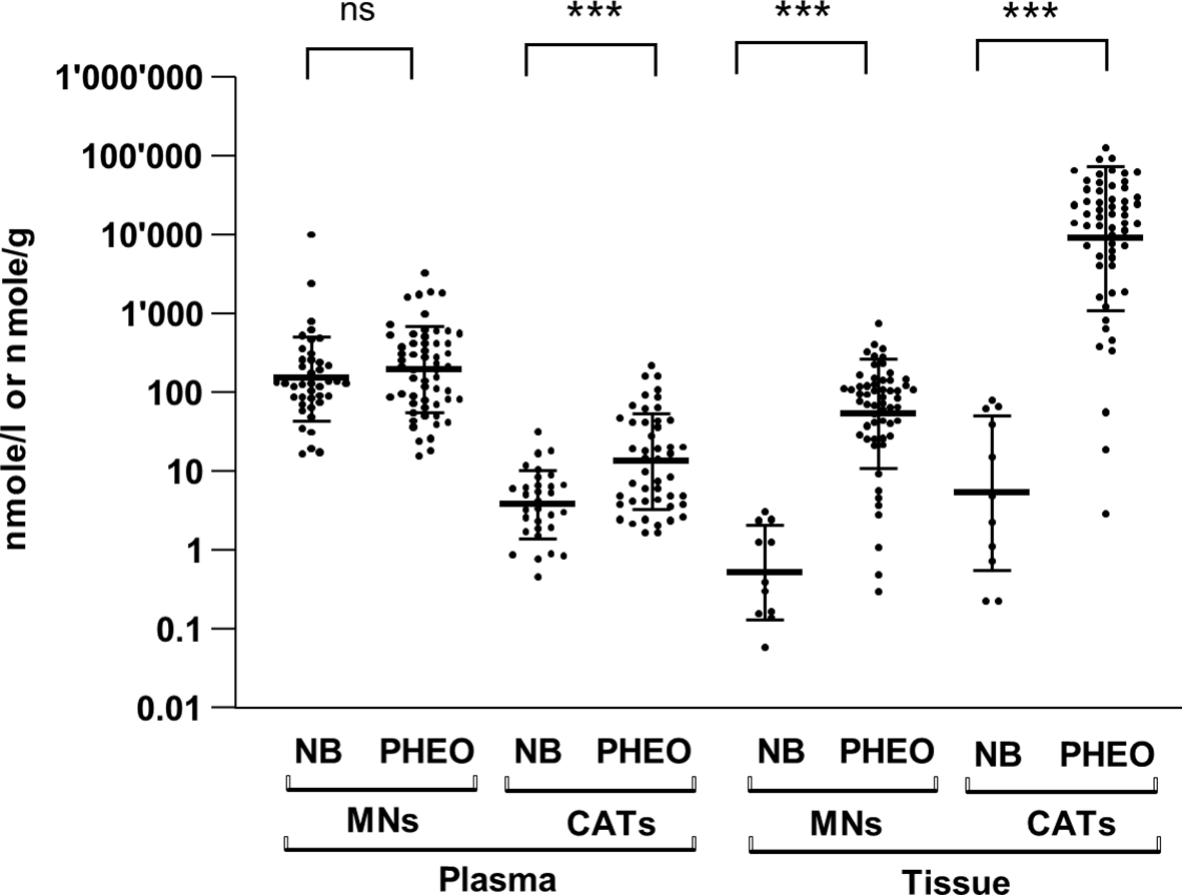
Plasma and tissue concentrations of CATs and MNs in NB and PHEO. Sum of CATs/MNs values and geo mean ± geo SD are plotted on a logarithmic scale and analyzed with a non-parametric Mann-Whitney test (non significant= ns, ***=*p*<0.001, ****=*p*<0.0001). Values are reported in the text. Number of patients for plasma values: MNs: NB (n=41), PHEO/PGL (n=57); CATs: NB (n=31), PHEO/PGL (n=47); and for tissue values: MNs: NB (n=11), PHEO/PGL (n=57); CATs: NB (n=11), PHEO/PGL (n=57). MNs concentrations represent total forms (free and SO_4_-conjugated forms) in plasma and free forms in tumors (no conjugated forms are detected in tumor). CATs values represent free forms in both plasma and tumors. Part of these values for NB (n=22/41 for plasma MNs, n= 21/31 for plasma CATs and n=10/11 for tissue values for CATs and MNs) were already published in another study comparing CATs values in human and mice with NB (17) (**Supplementary Table 1**).

Because NB secretes MNs into the blood, as does PHEO/PGL, it would be expected to produce and contain massive amounts of CATs and MNs. However, early studies reported a small amount of CATs stored in NB tissues, compared with PHEO/PGL (14). As accurate quantification of CATs metabolites was not technically feasible at the time, we measured CATs and MNs levels by UHPLC-MS/MS in tumor tissues and in plasma of NB and PHEO/PGL patients. In order to compare the biosynthesis and metabolism of CATs and MNs in NB and PHEO/PGL patients we first determined the total amount of CATs (DA, NE and E) and MNs (MT, NMN, MN) stored in both type of tumors and released into the blood. In plasma, we observed comparable values of MNs (geo. mean NB: 151.8 and PHEO/PGL: 195.4 nmol/l, 1.3x), in contrast to CATs that were 3.5 fold higher in PHEO/PGL than in NB (NB: 3.8 and PHEO/PGL: 13.5 nmol/l) (**Figure 1** and **Supplementary Figures 1A-B**). Although in plasma global MNs values were found in similar concentrations for NB and PHEO patients, MT levels were more elevated while MN levels were reduced in NB relative to PHEO. In tumor tissues, drastic reductions in MNs (-103x) and CATs (-1671x) concentrations were observed in NB as compared to PHEO/PGL, with 0.53 versus 54.9 nmol/g for MNs and 5.4 versus 8992 nmol/g for CATs, respectively (**Figure 1** and **Supplementary Figures 1C-D**). This confirms, in a large cohort of patients, the striking difference in CATs and MNs concentrations in NB versus PHEO/PGL.

### Chromaffin markers and neurosecretory vesicle content are reduced in NB compared with PHEO/PGL

**Figure 2:**
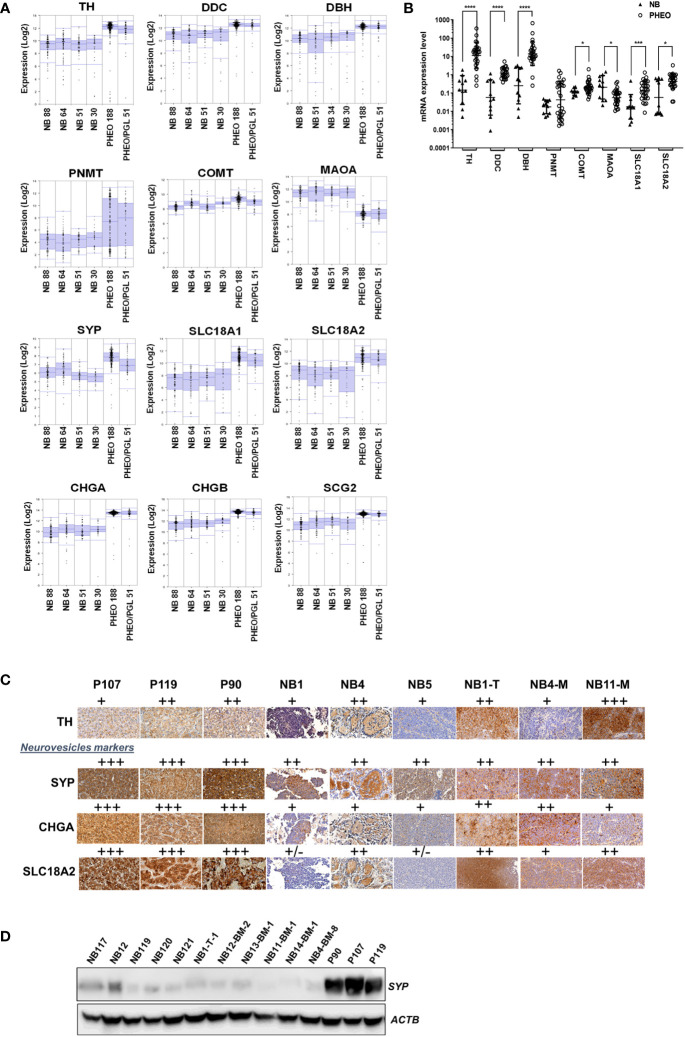
Expression level of genes involved in CATs metabolism and NV markers in NB and PHEO. **(A)** Expression levels (log2) of the indicated genes in 4 NB transcriptomic datasets (Versteeg n=88, Hiyama n= 51, Delattre n=34, Lastowska n=30) and 2 PHEO/PGL datasets (Favier n=188, and Korpershoek n=51) analyzed by microarray using the R2: Genomics Analysis and Vizualization Platform (http://r2.amc.nl, MegaSampler analysis: Human Genome U133, Plus 2.0; MAS5.0 data normalization). **(B)** mRNA quantification by RT-qPCR of the main enzymes involved in CATs metabolism and NV markers from NB (n=10) and PHEO/PGL (n=28) tissues. Individual values and geo means ± SD are plotted and analyzed with a non-parametric Mann-Whitney test. *p* values are reported only when statistically significant (<0.05), *=*p*<0.05, ***=*p*<0.001, ****=*p*<0.0001). The values for NB were already published in another study comparing primary NB and NB-PDX (17) (**Supplementary Table 1**). **(C)** Representative images of IHC staining the detection of TH, SYP, CHGA and SLC18A2 protein expression on three PHEO/PGL tissue: P90, P107, P119, three NB biopsies from patients: NB1, NB4 and NB5 and three NB-PDX: NB1-T-1, NB4-BM-8, NB11-BM-1. The results were semi-quantitatively scored (+, faint; ++, moderate; +++, strong). **(D)** Immunoblotting for the detection of SYP in tissue samples from five NB biopsies (4 after chemotherapy and one, sample NB-121 at the time of diagnosis), six NB-PDX : NB1-T-1, NB12-BM-2, NB13-BM-1, NB11-BM-1, NB14-BM-1, and NB4-BM-8, and from three PHEO/PGL: P90, P107, P119. Volumes loaded were 10µl from a 20% wt/vol extracts. ACTB was used as loading control.

The lower concentration of CATs detected in NB tissues compared with PHEO/PGL may result from reduced production of CATs, more efficient conversion to MNs, and/or a lower amount of neurosecretory vesicles (NVs) in the cytoplasm. In the latter scenario, newly synthesized DA is metabolized to MT by COMT and a fraction is converted to NE by DBH present in the rare NVs. NE is then metabolized to NMN in the cytoplasm and MT and NMN diffuse freely into the blood. To explore these hypotheses, we compared the expression levels of key enzymes involved in CAT metabolism (TH, DBH, DDC, PNMT, COMT, and MAOA) and NV markers in NB and PHEO/PGL in four NB primary tumor datasets and two PHEO/PGL series using the R2: Genomics Analysis and Visualization Platform (http://r2.amc.nl). We choose as NV markers in addition to SLC18A1/2, synaptophysin (SYP), which is an integral NV membrane protein, as well as chromogranin A and B (CHGA/B) and secretogranin 2 (SCG2), three prohormones co-released with CATs. These proteins are commonly used as general neuroendocrine markers in IHC analyses (18, 28–32). This *in silico* analysis in large tumor datasets demonstrated that the expression levels of TH, DDC, and DBH were reduced in NB compared to PHEO/PGL, inversely to MAOA levels. As previously demonstrated, PNMT levels were almost undetectable in NB and highly variable in PHEO/PGL due to the adrenergic and noradrenergic phenotypes of these tumors (16), while very similar levels of COMT were found in NB and PHEO. Regarding NV markers, the levels of SYP, SLC18A1/2, CHGA/B and SCG2 were also lower in NB compared to PHEO/PGL (**Figure 2A**). These data were confirmed by qPCR mRNA quantification in a cohort of tissues from NB and PHEO/PGL with again a strong decrease in TH, DDC, DBH expression levels in NB (**Figure 2B**).

We also validated these observations at the protein level, as PHEO/PGL tissues gave a stronger signal by IHC for TH and particularly for the vesicular markers SYP and SLC18A2 and the secretory granular marker CHGA compared with NB and NB-PDX tissues (**Figure 2C**). By immunoblotting, it was confirmed that SYP was highly expressed in PHEO/PGL while only a weak signal was detected in 5 NB and 6 NB-PDX biopsies (**Figure 2D**). It is noteworthy that NB-PDX tumors were recently described by our group as representing a reliable tool to study CAT metabolism in NB (17). Overall, these data suggest not only a reduced production of CATs but also a reduced amount of NV in NB compared to PHEO/PGL.

**Figure 3:**
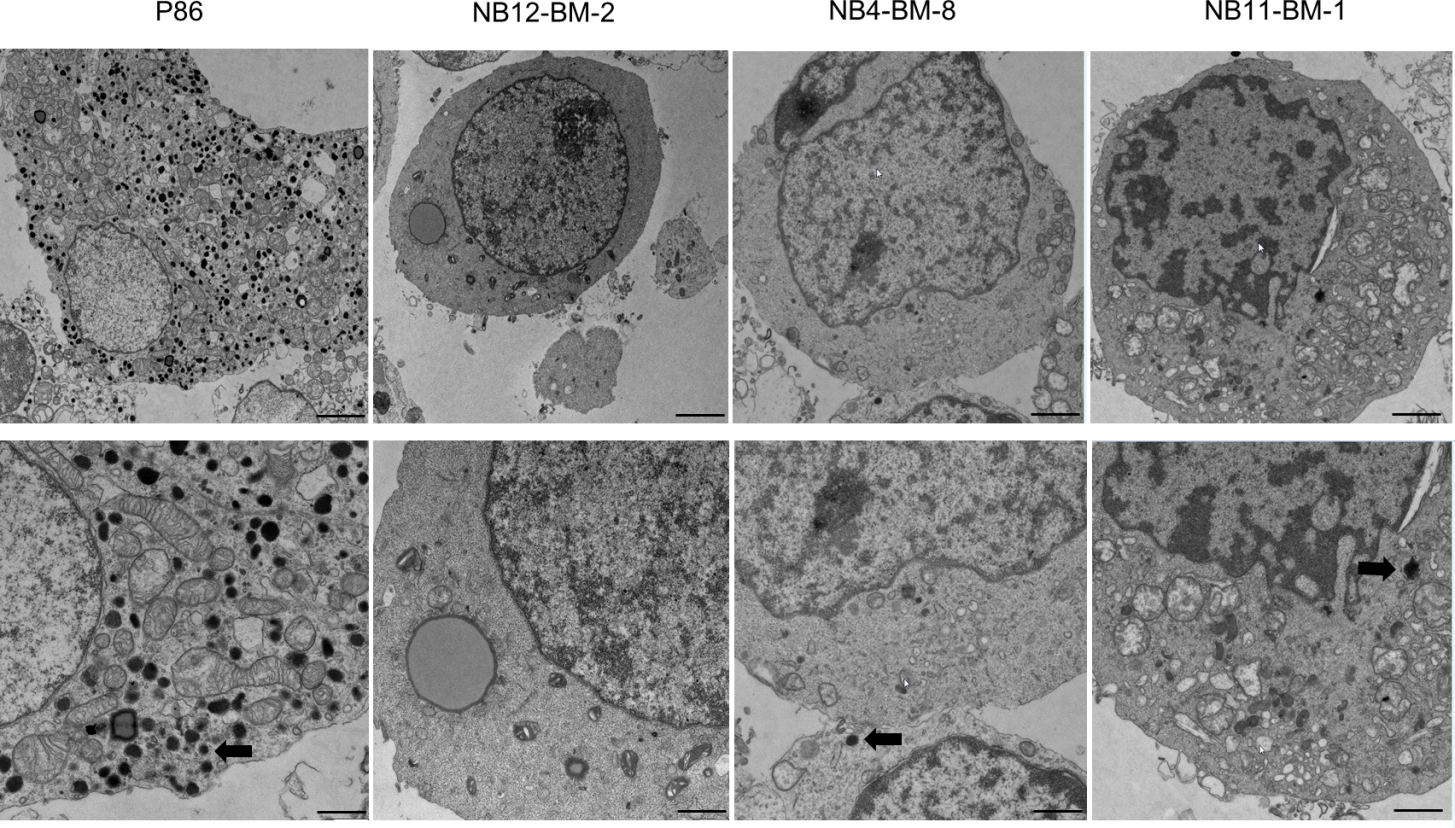
Analysis of the amount of neurosecretory vesicles in primary NB and PHEO cells by electron microscopy. Representative images of electron micrographs of PHEO/PGL and NB-PDX cells dissociated from primary tumor biopsies and xenografts, respectively. Lower panels: zoom of upper panels showing in details the cytoplasm. Thick arrows shows the electron dense content that correspond to CATs in neurosecretory vesicles. Scale bars: 1µm (upper panels, 1^st^ and 2^nd^ column), 2µm (upper panels, 3^rd^ and 4^th^ column) and 500nm (lower panels).

To directly confirm that these different expression levels of NV markers correlate with the amount of NV in the cytoplasm of NB and PHEO/PGL, we performed electron microscopy on isolated cells from PHEO/PGL biopsy and NB-PDX tissue. We observed and counted numerous densely nucleated vesicles corresponding to CATs storage NVs in PHEO (P86) cells with a mean ± SD of 124 ± 22.03 NVs/cellular field (n=20). This is in contrast to NB cells where the cytoplasm was almost devoid of NVs in all 3 cell types studied (mean ± SD for NB12-BM2: 1.1 ± 0.98 NV/cellular field (n=44), for NB4-BM-8: 0.8 ± 0.65 NV/cellular field (n=39), and for NB11-BM-1: 1.25 ± 1.47 NV/cellular field (n= 43) (**Figure 3**).

### Low amounts of CATs in the cytoplasm of NB cell lines and primary cells, and in PDX

**Figure 4:**
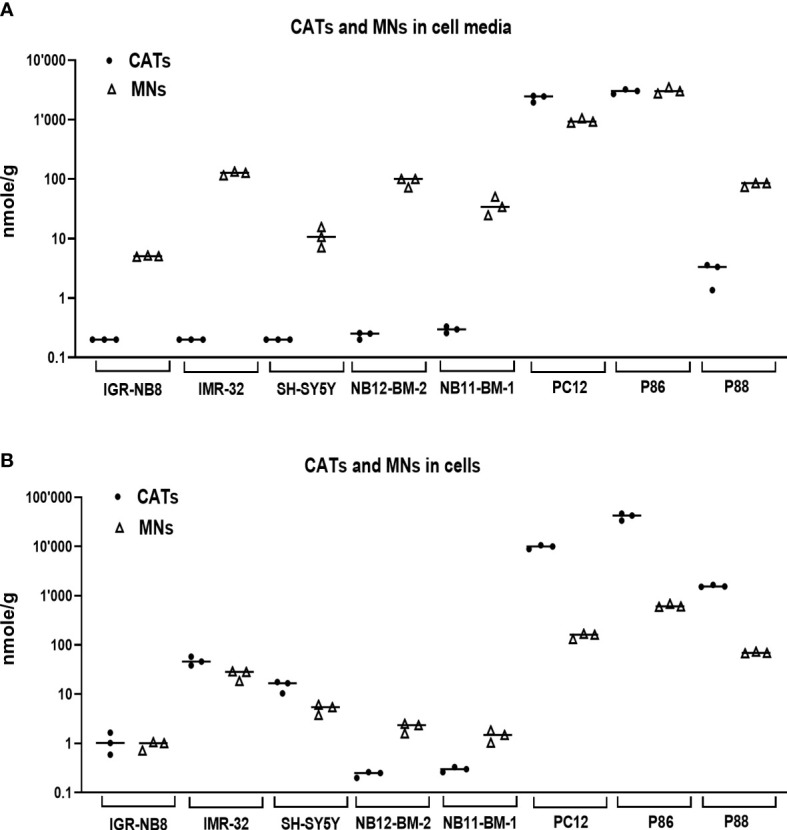
CATs and MNs concentrations in NB and PHEO/PGL cells *in vitro* and their culture media. **(A)** Quantification of sum of CATs (dots) and MNs (triangles) in cell culture medium (in nmol/g of protein in cell lysates) of three NB cells lines, two primary cell sample from NB-PDX (NB11-BM-1, NB12-BM-2), the PC12 cell lines, and two PHEO/PGL biopsies (P86 and P88). Assays were performed in triplicate and corresponding values are plotted in the graph with indication of the mean: IGR-NB8: 0.2 and 5.1 nmol/g for CATs and MNs respectively; IMR32: 0.2 and 125.2; SH-SY5Y: 0.2 and 11.1; NB12-BM-2: 0.24 and 91; NB11-BM-1: 0.3 and 36.4; PC12: 2300 and 957; P86: 2990 and 3087; P88: 2.8 and 81.3. Values under our limit of quantification were set at 0.025, 0.125 and 0.075 nmol/g for respectively E, NE and DA, which represent half of their lower limit of quantification (LLOQ). **(B)** Quantification of intracellular CATs and MNs (in nmol/g of protein) in the corresponding cell lysates from **(A)** Mean values: IGR-NB8: 1.1 and 0.9 nmol/g for CATs and MNs respectively; IMR32: 47.1 and 25.3; SH-SY5Y: 14.9 and 5.1; NB12-BM-2: 0.24 and 2.2; NB11-BM-1: 0.3 and 1.5; PC12: 9872 and 154.6; P86: 40910 and 634; P88: 1576 and 70.

Given the very low amounts of CATs and MNs metabolites stored in NB tissues (**Figure 1**), we sought to further investigate the molecular basis for this finding. Thus, the concentrations of CATs and MNs were measured in cell lysates and supernatants of 4 noradrenergic NB cell lines: IGR-NB8, SH-SY5Y, LAN-1, IMR32, and a PHEO/PGL cell line (PC12), as well as dissociated primary cells from two PHEO/PGL biopsies (P86, P88) and two NB-PDX (NB11-BM-1, NB12-BM-2) (17) cultured *in vitro*. We observed trace amounts near or below our limit of quantification for CATs in the culture medium of all NB cell lines and PDX-derived cells. This is in contrast to the substantial amounts of CATs detected in the cell medium of PC12 (mainly due to the concentration of DA) and PHEO/PGL primary cells (P86 and P88) (**Figure 4A**). The differences between NB and PHEO/PGL were even greater when considering the amounts of CATs and MNs stored in the cytoplasm of the cells (**Figure 4B**). Interestingly, NB primary cells (NB11-BM1 and NB12-BM2) had a higher MNs content than CATs, whereas the opposite ratio was observed for PHEO/PGL primary cells where a massive concentration of CATs was detected, giving further evidence that a significant amount of CATs is not stored inside NVs and available for processing into MNs in NB.

### Induction of NV genesis allows the NB cell line to protect and secrete CATs

**Figure 5:**
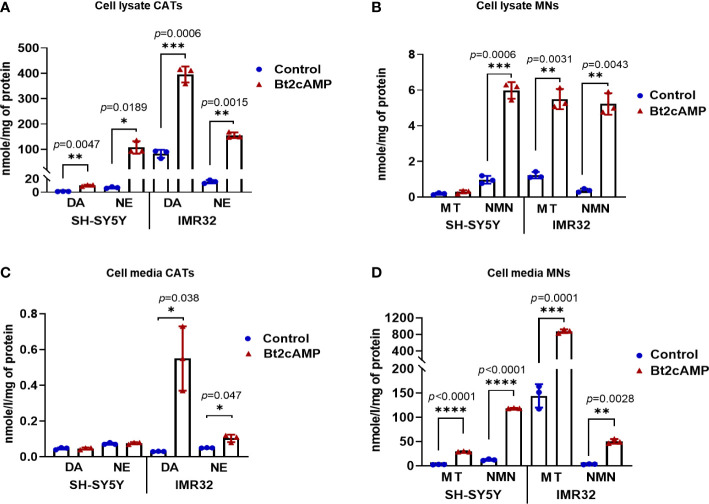
Induction of CATs synthesis through Bt2cAMP treatment of NB cells. Concentrations of CATs and MNs in SH-SY5Y and IMR32 cell lysates **(A, B)** and cell culture media **(C, D)** after 4 days with or without Bt2cAMP incubation. Assays were performed in triplicate and corresponding values are plotted in the graph with mean and SD. **(A)** CATs: SH-SY5Y: DA: 10.21 vs 1.14 nmol/g (treated vs control), NE: 107.5 vs 6.82 nmol/g; IMR32: DA: 395.5 vs 82.13 nmol/g, NE 155.43 vs 15.67 nmol/g, respectively. **(B)** MNs: SH-SY5Y: MT: 0.29 vs 0.19 nmol/g, NMN: 5.98 vs 0.97 nmol/g; IMR32: MT: 5.49 vs 1.22 nmol/g, NMN: 5.22 vs 0.37 nmol/g, respectively. **(C)** CATs: IMR32: DA: 0.55 vs 0.03 nmol/g, NE 0.1 vs 0.05 nmol/g, respectively **(D)** MNs: SH-SY5Y: MT: 29.37 vs 2.65 nmol/g, NMN: 118.75 vs 12.29 nmol/g; IMR32: MT: 877.89 vs 144.35 nmol/g, NMN: 50.49 vs 3.29 nmol/g. Individual values and means ± SD are plotted and analyzed with a Welch’s t-test. *p* values are reported only when statistically significant (<0.05), *=*p*<0.05, ***=*p*<0.001, ****=*p*<0.0001).

Although CATs are actively synthesized in NB cells, the scarcity of NVs prevents the protection of CATs from degradation by MAOA and COMT enzymes, resulting in pathological values for MNs in the blood of patients. This could also explains the very low amount of CATs measured in NB tissues and the normal values of CATs in the blood of most NB patients. To address this hypothesis, we therefore investigated whether an increase in NV synthesis correlated with an increase in CATs concentration and whether newly synthesized NVs were functional in terms of CATs secretion upon pharmacological stimulation. To this end, SH-SY5Y and IMR32 cells were treated with Bt2cAMP to induce differentiation into a noradrenergic phenotype, as previously described (33, 34). We observed an increase in intracellular DA and NE levels after 4 days of treatment compared to untreated cells. For SH-SY5Y: a 9-fold increase for DA and 15.8-fold increase for NE was recorded and for IMR32 the fold change was 4.8 and 9.9 for DA and NE, respectively, while no E was detected in both cell lines due to the absence of PNMT in NB cells (17) (**Figure 5A**). Intracellular concentrations of NMN and MT were also increased in both cell lines: SH-SY5Y: 1.5-fold for MT and 6.2-fold for NMN. For IMR32, the increase was 4.5-fold for MT and 13.8-fold for NMN (**Figure 5B**). In the incubation medium, very low levels of CATs were detected for SH-SY5Y cells with or without BT2cAMP, whereas differentiation induced an increase in CATs concentration for IMR32 cells (DA: 18.3x, NE: 2x). The levels of CATs released into the culture media were also very low compared with MNs, which were increased upon Bt2cAMP treatment (SH-SY5Y fold change: 11.1X for MT and 9.7X for NMN; and IMR32: 6.1X for MT and 15.3X for NMN) (**Figures 5C, D**).

**Figure 6:**
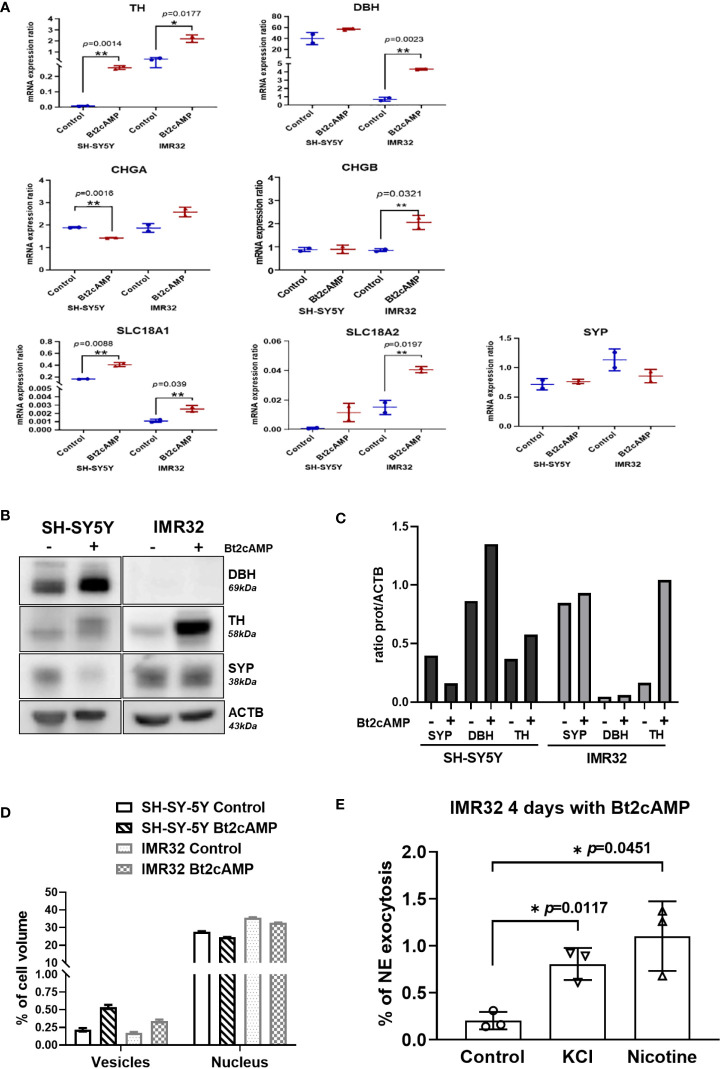
NV induction and exocytosis in cAMP treated cells. **(A)** mRNA quantification of TH, DBH, CHGA, CHGB, SLC18A1, SLC18A2 and SYP in SH-SY5Y and IMR32 cell lines treated without (Control) and with 500nM Bt2cAMP for 4 days. Fold change: TH: 42.6 in SH-SY5Y and 6 in IMR32, DBH: 1.42 in SH-SY5Y, 6.2 in IMR32, CHGA: 0.75 in SH-SY5Y and 1.38 in IMR32, CHGB: 1.5 in IMR32 cells, SLC18A1: 2.5 in SH-SY5Y and 2.3 IMR32; SLC18A2: 12.8 in SH-SY5Y and 2.7 in IMR32. Assays were performed in triplicate and individual values and means ± SD are plotted and analyzed with an unpaired t-test. *p* values are reported only when statistically significant (<0.05). *=*p*<0.05, **=*p*<0.01. **(B)** Immunoblotting for SYP, DBH and TH expression in cells treated as in **(A, C)** Quantification of immunoreactive signal normalized with ACTB protein expression using the Image J software. **(D)** Bare graph representing the counting of NVs (vesicles) and nucleus volume per cell using stereologic method on 2 fields of 120x120 µm per condition (approx. 30 and 40 cells/fields for SH-SY5Y and IMR32 cells, respectively). **(E)** Quantification of NE following chemical stimuli (56mM KCl or 100 µM nicotine) from IMR32 cells incubated 4 days with 500 nM Bt2cAMP. Values are expressed in % of NE found in cell medium regards to total NE (cytoplasmic plus cell medium concentrations). NE: values for control, KCl and nicotine: 0.2, 0.81 and 1.1% respectively. Assays were performed in triplicate and individual values and means ± SD are plotted and analyzed with a Welch’s t-test. *=*p*<0.05.

The molecular basis of the NB cell response to differentiation was then analyzed by measuring the mRNA expression levels of TH and DBH and NV markers (SYP, CHGA/B, SLC18A1/2) (**Figure 6A**), as well as the protein levels of TH, DBH, and SYP (**Figures 6B, C**). The expression of TH was significantly increased in both cell lines, which was confirmed at the protein level. The level of DBH mRNA was significantly increased in the IMR32 line after Bt2cAMP treatment, but DBH protein expression was below the limit of detection. This explains the low amount of NE and NMN produced by this cell line compared with DA and MT (**Figures 5A, B**). Overall, mRNA expression levels of NV markers were higher in treated cells compared with controls, with the exception of CHGA mRNA, which was slightly reduced by Bt2cAMP treatment in SH-SY5Y cells, and SYP protein level, which was reduced in SH-SY5Y cells.

Because several NV markers were upregulated after pharmacological differentiation of both cell lines, we performed electron microscopy studies to morphologically assess a possible increase in NV size and/or concentration in the cell cytoplasm. Using a stereological method for NV quantification (see Materials and Methods), we measured a 2- and 2.5-fold increase in NV volume in IMR32 and SH-SY5Y cell lines, respectively, after treatment with 500 nM Bt2-cAMP for 4 days compared with controls (**Figure 6D**, **Supplementary Figure 2**).

We next investigated whether differentiation led to proper internalization of CATs into newly formed NVs, and thus whether differentiated cells could respond to exocytosis stimuli, such as KCl or nicotine (25). Because SH-SY5Y cells do not produce enough CATs to be reliably quantified in the cell medium, even after pharmacological differentiation, the tests were performed on IMR32 cells only. The percentage of NE exocytosis was statistically significantly increased after treatment with KCl or nicotine by 4 and 5.4-fold, respectively (**Figure 6E**).

## Discussion

Both NB and PHEO/PGL are non-epithelial neuroendocrine neoplasms arising from sympathoadrenal tissues. Plasma MNs represent reliable biomarkers for these CATs-producing tumors (19, 35), however, NB tissues have been shown to contain low amounts of CATs, contrasting with the massive concentrations in PHEO/PGL (14, 36). In this study, we performed an extensive comparative analysis of the biosynthesis, metabolism, and storage of CATs and MNs in NV for both tumor types. First, using UHPLC-MS/MS to quantify the metabolites of CATs and MNs in tumors and plasma, we showed that intratumoral concentrations of CATs in NB are several orders of magnitude lower than those in PHEO/PGL, confirming early studies performed with less sensitive methods (14, 36). Furthermore, we demonstrated that the amount of MNs is also greatly reduced in NB compared to PHEO/PGL tumor tissues. In contrast, in plasma, we observed that CATs were slightly higher in PHEO/PGL-affected individuals, whereas overall MNs levels were comparable between NB and PHEO/PGL patients. However, the relative profiles of MNs were nevertheless distinct in the plasma of the two tumor types, with higher concentrations of MT and reduced levels of MN in NB compared to PHEO/PGL, as expected due to the noradrenergic phenotype of NB (17).

Next, we analyzed and compared the expression levels of various chromaffin markers, including enzymes involved in CATs biosynthesis (TH, DDC, DBH, PNMT) and transformation (COMT and MAOA), as well as markers of CATs storage vesicles (SYP, SLC18A1/2, CHGA, CHGB and SCG2) between NB and PHEO/PGL. Our data revealed reduced expression of most of these markers in NB, with the exception of MAOA and COMT. Low MAOA expression in PHEO/PGL compared to healthy tissue has been previously reported (12), but the consequence of this higher expression in NB has not been studied in detail. As NB and PHEO express a similar amount of COMT, this may explain why MNs are also reliable biomarkers for NB despite their reduced capacity for CATs production and storage. Interestingly, this set of genes, as well as other chromaffin markers, were identified as genes specifically overexpressed in NB and PHEO/PGL compared to various cancerous and normal tissue settings in a large-scale *in silico* analysis of transcriptomic data (37). However, although their differential expression analysis between NB and PHEO/PGL confirmed the downregulation of many chromaffin markers in NB (except MAOA: overexpressed, COMT: not differentially expressed), the authors did not report this fact in their manuscript.

The low expression levels of chromaffin markers in NB as a CATs-producing tumor suggest a reduced capacity for CATs synthesis and storage compared with PHEO/PGL. Here, we also demonstrate that the low amount of CATs stored in the cytoplasm of NB cells is mainly due to a low amount of NVs produced in the majority of tumor cells, as illustrated by electron microscopy analysis showing the paucity of NVs identified in NB cells, which is in accordance with previous studies (14, 18, 38).

One of the reasons for the low concentration of NV could come from a downregulation of several proteins involved in NV biogenesis with notably myosin 1b and F-actin (39), or as more recently demonstrated CHGA (40, 41). It is interesting to note that this last protein is present in high concentration in PHEO and in lower concentration in NB, although CHGA represents a histochemical marker of these two types of tumors. Thus, a low concentration of CHGA in NB could be one of the reasons rather than a consequence of the lower number of NV in NB compared to PHEO.

A recent study in primary PHEO cells demonstrated an increase in many proteins involved in vesicular exocytosis or CATs synthesis as well as a higher number of exocytotic events in PHEOs compared with chromaffin cells at the single cell level for the same stimulation (42). This suggests in cells secreting high concentrations of CATs an increase in the number of NVs rather than an increase in the storage capacity of each NV. In this case, a larger storage of CATs will necessarily imply more of these NVs in the cytoplasm while an insufficient number will imply rapid degradation to MNs and/or DHPG. It is noteworthy that NB and PHEO express similar amounts of COMT. This may explain the high level of MNs in NB despite a low amount of NVs, as the CATs that escape from the few NVs are efficiently transformed into MNs.

Whereas PHEO/PGLs derive from fully differentiated chromaffin cells of the adrenal medulla, pediatric solid tumors, such as NB, are thought to arise from developmental defects affecting the normal sympathoadrenergic differentiation and maturation program (43). Therefore, the reduced amount of noradrenergic markers and neurosecretory vesicles observed in NB compared to PHEO/PGL may result from blockade in the differentiation program in sympatho-adrenal progenitors at the origin of NB, although partial dedifferentiation cannot be excluded. Indeed, recent single-cell transcriptomic analysis of primary NBs and adrenal glands confirmed that NBs are predominantly composed of cells with transcriptional signatures of adrenal neuroblasts/sympathoblasts, which are distinct from adrenal chromaffin cells, although a small proportion of chromaffin cells have been identified in several high-risk NBs (44, 45).

Differentiation with retinoic acid (13-cis RA) is part of the maintenance phase of current treatment protocols for high-risk NB. It has been shown to inhibit cell proliferation and induce differentiation characterized by increased expression of various neuronal markers and neurite outgrowth *in vitro* (46, 47). However, retinoic acid has been shown to be ineffective in inducing differentiation to a noradrenergic/chromaffin phenotype, as the CATs concentration remains low (48, 49). A recent study provided a mechanistic explication for these observations, as it was that retinoic treatment reprograms the enhancer landscape and alters the noradrenergic core regulatory circuitry (NOR-CRC) of NB cells, by reducing the expression of the transcription factors Phox2b, GATA3, and MYCN (50). In contrast, Bt2cAMP has been shown to induce noradrenergic differentiation in NB cell lines, as evidenced by increased CATs synthesis and TH expression (33, 34, 49). Here, our functional studies using Bt2cAMP as a differentiating agent resulted in an increased in NV number and volume as well as upregulation of the NV marker SLC18A1/2. Bt2cAMP also induced the synthesis of TH, the rate-limiting enzyme for CATs synthesis, and DBH, leading to the concomitant increase in CATs and MNs biosynthesis, as revealed by their higher concentrations in the cell cytoplasm and in the culture media. Furthermore, we demonstrated that these newly produced NV mediated by Bt2cAMP were fully functional as exocytosis stimuli led to an increase in CATs in the cell media.

A limitation of this study is that our cohort of NB and PHEO/PGL samples used for the measurement of CATs and MNs in plasma and tumor tissue, as well as NV detection, is only partially complete (some samples were not available for metabolite, mRNA, or protein quantification because of material scarcity), as detailed in **Supplementary Tables 1A, B**. For a similar reason, electron microscopy detection of NVs for NB was performed in PDX-derived tumor cells and cell lines rather than using primary tumor-derived cells. However, our NB-PDX models have been shown to closely mimic primary NB in terms of CATs synthesis, metabolism and storage (17).

From a clinical point of view, our data may explain at the cellular and molecular levels the low incidence of hypertension recorded in children with NB, which contrasts with the hypertension usually associated with PHEO/PGL. Indeed, NB cells containing rare NV cannot efficiently store E and NE and release them into plasma by exocytosis. This differs from PHEO/PGL, where hypertension is a classic symptom of the disease, because of the massive and episodic exocytosis of CATs into the bloodstream. As an extension of this study, it would be interesting to evaluate whether, in NB, the amount of intratumoral CATs and NV is increased for the rare NB patients diagnosed with tumor-induced hypertension without renal artery compression.

In conclusion, we demonstrated that the metabolism of CATs in NB differs from that well-characterized in PHEO/PGL, with low amounts of chromaffin and NV markers in NB, resulting in low intratumoral and plasma concentrations. Thus, in NB cells, DA synthesized in the cytoplasm by DDC is immediately available for conversion to DOPAC *via* MAOA, to MT *via* COMT, or to NE *via* DBH in NV. Because cytoplasmic NE and DA are available for MAOA and COMT catalysis, DHPG, DOPAC, NMN, and MT are therefore produced in large quantities and these metabolites subsequently diffuse into the bloodstream (10). DHPG and DOPAC are not specific tumor markers due to their synthesis in other tissues and cell types, so they were not measured in this study. This scenario would explain the increase in plasma NMN and MT concentrations, which are used as biomarkers of NB (3). Overall, our study also updated and detailed with modern technologies the early observations on cellular CAT metabolism in NB (18, 36).

## Data availability statement

The original contributions presented in the study are included in the article/**Supplementary Material**. Further inquiries can be requested to the corresponding author.

## Ethics statement

The protocol for this study was approved by the local Ethics committee and all families signed an informed consent. Animal experimentation protocols were approved by the Swiss Federal Veterinary Office.

## Author contributions

AM-M and KA conceived the study. KA, KB, SU, PE, AM-M and JD performed the experiments. MP, EG, AM-M and KA interpreted the data and wrote the manuscript. All authors contributed to the article and approved the submitted version.

## Funding

The FORCE foundation supported the salary of lab technician involved in part of this project. Open access funding was provided by the University of Lausanne.

## Acknowledgments

We thank Mrs C. Centeno, C. Seghezzi and M. Dunand for excellent technical help for metabolites quantification.

## Conflict of interest

The authors declare that the research was conducted in the absence of any commercial or financial relationships that could be construed as a potential conflict of interest.

## Publisher’s note

All claims expressed in this article are solely those of the authors and do not necessarily represent those of their affiliated organizations, or those of the publisher, the editors and the reviewers. Any product that may be evaluated in this article, or claim that may be made by its manufacturer, is not guaranteed or endorsed by the publisher.
