# Supplementary material for: Low number of neurosecretory vesicles in neuroblastoma impairs massive catecholamine release and prevents hypertension
Source: Front Endocrinol (Lausanne). 2022 Dec 1;13:1027856. doi: 10.3389/fendo.2022.1027856 (PMC9751011; doi:10.3389/fendo.2022.1027856)
Supplement: Supplementary file 1 [file DataSheet_1.pdf]

Supplementary Material

Supplementary Figures and Tables

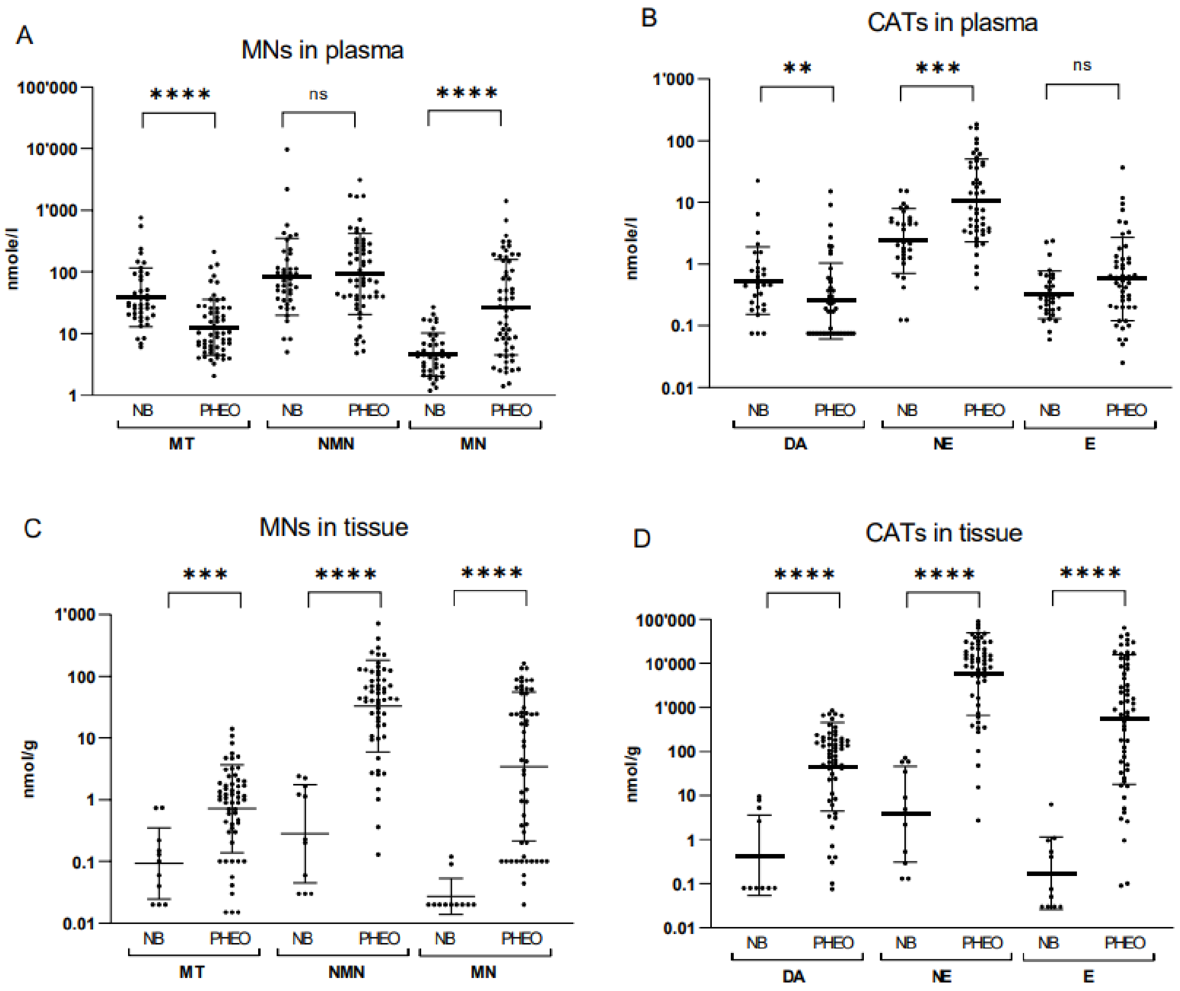

Supplementary Figure 1. (A) Individual values for MT, NMN and MN from Figure 1. Mean values for: MT NB and PHEO/PGL: 29.42 and 10.11 nmol/l respectively. NMN: 72.71 and 74.14. MN: 4.29 and 25.26. **(B)** As in A: individual values for DA, NE and E for NB and PHEO/PGL: DA: 0.47 and 0.19 respectively, NE: 2.27 and 7.78, E: 0.28 and 0.54. **(C)** Individual values for MT, NMN and MN in tissue for NB and PHEO/PGL respectively: MT: 0.1 and 1.07, NMN: 0.23 and 44.23, MN: 0.02 and 7.4. **(D)** As in C: individual values for CATs: DA: 0.08 and 101.8, NE: 4.87 and 11020, E: 0.075 and 901.8. Part of these values for NB (n=22/41 for plasma MNs and n= 11/31 for plasma CATs and n=10/11 for tissue values) were already published in another study comparing CATs values in human and mice with NB [16]. CATs/MNs values and geo mean ± geo SD are plotted on a logarithmic scale

and analyzed with a non-parametric Mann-Whitney test (non significant=ns, **=p<0.01, ***=p<0.001, ****=p<0.0001).

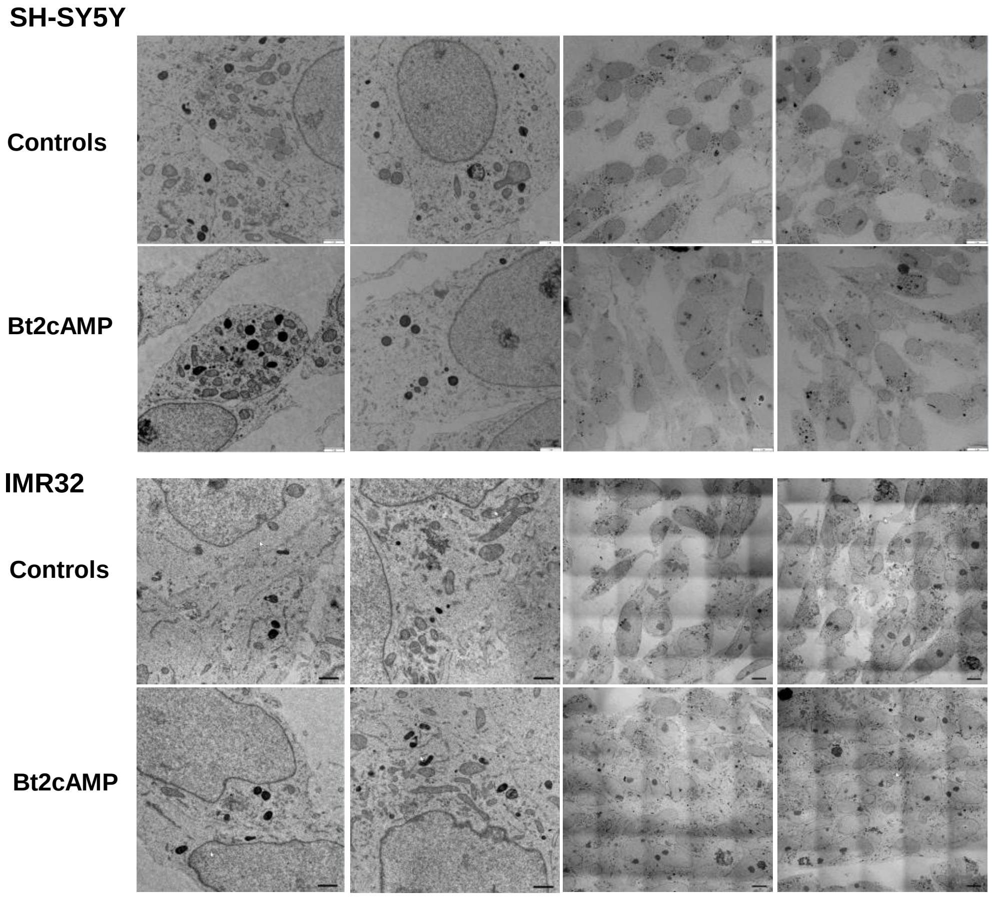

Supplementary Figure 2. Representative images of electron micrographs of SH-SY5Y and IMR32 cell lines with and without (controls) incubation of 500uM of Bt2cAMP Scale bars: 1 µm (1st and 2nd column) and 7µm (3rd and 4th column).

Supplementary Table 1A

PHEO/PGL	Plasma (Fig.1)		Tissue (Fig.1)						
#patient	MNs	CATs	MNs	CATs	qPCR (Fig. 2B)	IHC (Fig. 2C)	WB (Fig. 2D)	EM (Fig 3)	CATs/MNs (Fig. 4)
P01	✓	n.a.	✓	✓	✓	n.a.	n.a.	n.a.	n.a.
P02	✓	n.a.	✓	✓	✓	n.a.	n.a.	n.a.	n.a.
P03	✓	✓	✓	✓	✓	n.a.	n.a.	n.a.	n.a.
P04	✓	✓	✓	✓	✓	n.a.	n.a.	n.a.	n.a.
P05	✓	n.a.	✓	✓	✓	n.a.	n.a.	n.a.	n.a.
P06	✓	n.a.	✓	✓	✓	n.a.	n.a.	n.a.	n.a.
P07	✓	n.a.	✓	✓	✓	n.a.	n.a.	n.a.	n.a.
P08	✓	✓	✓	✓	✓	n.a.	n.a.	n.a.	n.a.
P09	✓	✓	✓	✓	✓	n.a.	n.a.	n.a.	n.a.
P10	✓	✓	✓	✓	✓	n.a.	n.a.	n.a.	n.a.
P11	✓	n.a.	✓	✓	✓	n.a.	n.a.	n.a.	n.a.
P12	✓	✓	✓	✓	✓	n.a.	n.a.	n.a.	n.a.
P13	✓	✓	✓	✓	✓	n.a.	n.a.	n.a.	n.a.
P14	n.a.	n.a.	n.a.	n.a.	✓	n.a.	n.a.	n.a.	n.a.
P15	✓	n.a.	✓	✓	✓	n.a.	n.a.	n.a.	n.a.
P16	✓	✓	✓	✓	n.a.	n.a.	n.a.	n.a.	n.a.
P17	✓	n.a.	✓	✓	n.a.	n.a.	n.a.	n.a.	n.a.
P18	✓	✓	✓	✓	✓	n.a.	n.a.	n.a.	n.a.
P19	✓	n.a.	✓	✓	✓	n.a.	n.a.	n.a.	n.a.
P20	✓	✓	✓	✓	✓	n.a.	n.a.	n.a.	n.a.
P21	✓	n.a.	✓	✓	✓	n.a.	n.a.	n.a.	n.a.
P22	✓	✓	✓	✓	✓	n.a.	n.a.	n.a.	n.a.
P23	✓	✓	✓	✓	✓	n.a.	n.a.	n.a.	n.a.
P24	✓	✓	✓	✓	✓	n.a.	n.a.	n.a.	n.a.
P25	n.a.	n.a.	n.a.	n.a.	✓	n.a.	n.a.	n.a.	n.a.
P28	✓	✓	✓	✓	n.a.	n.a.	n.a.	n.a.	n.a.
P29	✓	✓	✓	✓	n.a.	n.a.	n.a.	n.a.	n.a.
P30	✓	✓	✓	✓	✓	n.a.	n.a.	n.a.	n.a.
P31	✓	✓	✓	✓	n.a.	n.a.	n.a.	n.a.	n.a.
P32	✓	✓	✓	✓	n.a.	n.a.	n.a.	n.a.	n.a.
P33	✓	✓	✓	✓	n.a.	n.a.	n.a.	n.a.	n.a.
P34	✓	✓	✓	✓	n.a.	n.a.	n.a.	n.a.	n.a.
P35	✓	✓	✓	✓	n.a.	n.a.	n.a.	n.a.	n.a.
P36	✓	✓	✓	✓	✓	n.a.	n.a.	n.a.	n.a.
P37	✓	✓	✓	✓	✓	n.a.	n.a.	n.a.	n.a.
P38	✓	✓	✓	✓	✓	n.a.	n.a.	n.a.	n.a.
P39	n.a.	n.a.	n.a.	n.a.	✓	n.a.	n.a.	n.a.	n.a.
P40	✓	✓	✓	✓	n.a.	n.a.	n.a.	n.a.	n.a.
P41	✓	✓	✓	✓	n.a.	n.a.	n.a.	n.a.	n.a.
P42	✓	✓	✓	✓	n.a.	n.a.	n.a.	n.a.	n.a.
P44	✓	✓	✓	✓	n.a.	n.a.	n.a.	n.a.	n.a.
P45	✓	✓	✓	✓	n.a.	n.a.	n.a.	n.a.	n.a.
P46	✓	✓	✓	✓	n.a.	n.a.	n.a.	n.a.	n.a.
P47	✓	✓	✓	✓	n.a.	n.a.	n.a.	n.a.	n.a.
P48	✓	✓	✓	✓	n.a.	n.a.	n.a.	n.a.	n.a.
P49	✓	✓	✓	✓	n.a.	n.a.	n.a.	n.a.	n.a.
P50	✓	✓	✓	✓	n.a.	n.a.	n.a.	n.a.	n.a.
P51	✓	✓	✓	✓	n.a.	n.a.	n.a.	n.a.	n.a.
P52	✓	✓	✓	✓	n.a.	n.a.	n.a.	n.a.	n.a.
P53	✓	✓	✓	✓	n.a.	n.a.	n.a.	n.a.	n.a.
P54	✓	✓	✓	✓	n.a.	n.a.	n.a.	n.a.	n.a.
P55	✓	✓	✓	✓	n.a.	n.a.	n.a.	n.a.	n.a.
P72	✓	✓	✓	✓	n.a.	n.a.	n.a.	n.a.	n.a.
P73	✓	✓	✓	✓	n.a.	n.a.	n.a.	n.a.	n.a.
P79	✓	✓	✓	✓	n.a.	n.a.	n.a.	n.a.	n.a.
P86	n.a.	n.a.	n.a.	n.a.	n.a.	n.a.	n.a.	✓	✓
P88	n.a.	n.a.	n.a.	n.a.	n.a.	n.a.	n.a.	n.a.	✓
P89	✓	✓	✓	✓	n.a.	n.a.	n.a.	n.a.	n.a.
P90	✓	✓	✓	✓	n.a.	✓	✓	n.a.	n.a.
P107	✓	✓	✓	✓	n.a.	✓	✓	n.a.	n.a.
P118	✓	✓	✓	✓	n.a.	n.a.	n.a.	n.a.	n.a.
P119	✓	✓	✓	✓	n.a.	✓	✓	n.a.	n.a.

Supplementary Table 1B

NB	Plasma (Fig.1)		Tissue (Fig.1)					NB-PDX				
#patient	MNs	CATs	MNs	CATs	qPCR (Fig. 2B)	IHC (Fig. 2C)	WB (Fig. 2D)	name	IHC (Fig. 2C)	WB (Fig. 2D)	EM (Fig. 3)	CATs/MNs (Fig. 4)
NB1	✓	n.a.	n.a.	n.a.	n.a.	✓	n.a.	NB1-T-1	✓	✓	n.a.	n.a.
NB3	✓(*)	✓(*)	✓(*)	✓(*)	n.a.	n.a.	✓					
NB4	n.a.	n.a.	n.a.	n.a.	n.a.	✓	n.a.	NB4-BM-8	✓	✓	✓	n.a.
NB5	n.a.	n.a.	n.a.	n.a.	n.a.	✓	n.a.					
NB6	✓	n.a.	✓	✓	✓(*)	n.a.	✓					
NB7	✓(*)	n.a.	n.a.	n.a.	n.a.	n.a.	n.a.					
NB8	✓(*)	✓(*)	n.a.	n.a.	n.a.	n.a.	n.a.					
NB9	✓(*)	✓(*)	n.a.	n.a.	n.a.	n.a.	n.a.					
NB10	✓(*)	✓(*)	n.a.	n.a.	n.a.	n.a.	n.a.					
NB11	✓(*)	✓(*)	n.a.	n.a.	n.a.	n.a.	n.a.	NB11-BM-1	✓	✓	✓	✓
NB12	n.a.	n.a.	n.a.	n.a.	n.a.	n.a.	n.a.	NB12-BM-2	✓	n.a.	✓	✓
NB13	n.a.	n.a.	n.a.	n.a.	n.a.	n.a.	n.a.	NB13-BM-1	✓	n.a.	n.a.	n.a.
NB14	n.a.	n.a.	n.a.	n.a.	n.a.	n.a.	n.a.	NB14-BM-1	✓	n.a.	n.a.	n.a.
NB100	n.a.	n.a.	✓(*)	✓(*)	✓(*)	n.a.	n.a.					
NB101	n.a.	n.a.	✓(*)	✓(*)	✓(*)	n.a.	n.a.					
NB102	n.a.	n.a.	✓(*)	✓(*)	✓(*)	n.a.	n.a.					
NB103	✓(*)	✓(*)	✓(*)	✓(*)	✓(*)	n.a.	n.a.					
NB106	n.a.	n.a.	n.a.	n.a.	✓(*)	n.a.	n.a.					
NB107	n.a.	n.a.	n.a.	n.a.	✓(*)	n.a.	n.a.					
NB108	n.a.	n.a.	n.a.	n.a.	✓(*)	n.a.	n.a.					
NB109	n.a.	n.a.	n.a.	n.a.	✓(*)	n.a.	n.a.					
NB110	n.a.	n.a.	n.a.	n.a.	✓(*)	n.a.	n.a.					
NB111	✓	✓	✓(*)	✓(*)	n.a.	n.a.	n.a.					
NB112	✓	✓	✓(*)	✓(*)	n.a.	n.a.	n.a.					
NB113	n.a.	n.a.	✓(*)	✓(*)	n.a.	n.a.	n.a.					
NB114	✓(*)	✓(*)	✓(*)	✓(*)	✓(*)	n.a.	n.a.					
NB116	✓(*)	✓(*)	✓(*)	✓(*)	n.a.	n.a.	n.a.					
NB117	n.a.	n.a.	n.a.	n.a.	n.a.	n.a.	✓					
NB119	n.a.	n.a.	n.a.	n.a.	n.a.	n.a.	✓					
NB120	n.a.	n.a.	n.a.	n.a.	n.a.	n.a.	✓					
NB121	✓(*)	✓(*)	n.a.	n.a.	n.a.	n.a.	n.a.					
NB122	✓(*)	✓(*)	n.a.	n.a.	n.a.	n.a.	n.a.					
NB123	✓(*)	✓(*)	n.a.	n.a.	n.a.	n.a.	n.a.					
NB124	✓(*)	✓(*)	n.a.	n.a.	n.a.	n.a.	n.a.					
NB125	✓	n.a.	n.a.	n.a.	n.a.	n.a.	n.a.					
NB126	✓(*)	✓(*)	n.a.	n.a.	n.a.	n.a.	n.a.					
NB127	✓	n.a.	n.a.	n.a.	n.a.	n.a.	n.a.					
NB128	✓	n.a.	n.a.	n.a.	n.a.	n.a.	n.a.					
NB129	✓(*)	✓(*)	n.a.	n.a.	n.a.	n.a.	n.a.					
NB130	✓	✓	n.a.	n.a.	n.a.	n.a.	n.a.					
NB131	✓	✓	n.a.	n.a.	n.a.	n.a.	n.a.					
NB132	✓(*)	✓(*)	n.a.	n.a.	n.a.	n.a.	n.a.					
NB133	✓	✓	n.a.	n.a.	n.a.	n.a.	n.a.					
NB134	✓(*)	✓(*)	n.a.	n.a.	n.a.	n.a.	n.a.					
NB135	✓(*)	✓(*)	n.a.	n.a.	n.a.	n.a.	n.a.					
NB136	✓	✓	n.a.	n.a.	n.a.	n.a.	n.a.					
NB137	✓	n.a.	n.a.	n.a.	n.a.	n.a.	n.a.					
NB138	✓(*)	✓(*)	n.a.	n.a.	n.a.	n.a.	n.a.					
NB139	✓(*)	✓(*)	n.a.	n.a.	n.a.	n.a.	n.a.					
NB140	✓	✓	n.a.	n.a.	n.a.	n.a.	n.a.					
NB141	✓	✓	n.a.	n.a.	n.a.	n.a.	n.a.					
NB142	✓(*)	✓(*)	n.a.	n.a.	n.a.	n.a.	n.a.					
NB143	✓	n.a.	n.a.	n.a.	n.a.	n.a.	n.a.					
NB144	✓	✓	n.a.	n.a.	n.a.	n.a.	n.a.					
NB145	✓	n.a.	n.a.	n.a.	n.a.	n.a.	n.a.					
NB146	✓(*)	✓(*)	n.a.	n.a.	n.a.	n.a.	n.a.					
NB147	✓	✓	n.a.	n.a.	n.a.	n.a.	n.a.					
NB148	✓	n.a.	n.a.	n.a.	n.a.	n.a.	n.a.					

Supplementary Table 1. A-B List of PHEO/PGL and NB patient samples used in this study. Samples marked with an asterisk (*) were published in the context of another study comparing primary NB and NB-PDX [17], n.a.: sample not available.

Supplementary Table 2

PHEO/PGL	Clinical details							
#patients	Age	Gender	Localization	Malignant Y/N	Hypertension Y/N	PHEO/PGL	Known mutation	Clusters
P01	22	F	L	N	N	PHEO	RET	Kinase signaling
P02	62	F	L	N	Y	PHEO	Sp, no screening	n.a.
P03	54	F	R	N	Y	PHEO	Sp, no screening	n.a.
P04	44	M	R	N	Y	PHEO	Sp, (RET, SDH-B,-C, VHL negative)	n.a.
P05	67	M	R	N	Y	PHEO	Sp, no screening	n.a.
P06	68	F	L	Y	Y	PHEO	Sp, (RET, SDH-B negative)	n.a.
P07	72	F	Abd	N	Y	PGL	Sp, no screening	n.a.
P08	78	F	R	N	Y	PHEO	Sp, (RET, SDH-B negative)	n.a.
P09	40	M	L	N	Y	PHEO	Sp, no screening	n.a.
P10	58	M	L	Y	Y	PHEO	Sp, (RET, VHL, SDH-B,-C,-D negative)	n.a.
P11	26	M	Abd	N	Y	PGL	SDH-B	Pseudohypoxia
P12	53	M	Abd	N	Y	PGL	Sp, (RET, VHL, SDH-B,-C,-D, MAX negative)	n.a.
P13	65	M	L	N	Y	PHEO	Sp, no screening	n.a.
P14	49	F	L	N	Y	PHEO	Sp, no screening	n.a.
P15	35	M	L	N	Y	PHEO	Sp, (RET, VHL, SDH-B, SDH-D negative)	n.a.
P16	21	F	Abd	Y	Y	PGL	SDH-B	Pseudohypoxia
P17	21	M	L	N	Y	PHEO	RET	Kinase signaling
P18	44	F	L	N	N	PHEO	RET	Kinase signaling
P19	23	M	R	N	Y	PHEO	Sp, (RET, VHL, SDH-B,-D negative)	n.a.
P20	30	F	R	N	Y	PHEO	NF1	Kinase signaling
P21	42	F	R	N	N	PHEO	RET	Kinase signaling
P22	33	M	Paraaortal R	Y	n.a.	PGL	Sp, (RET, VHL, SDH-B,-C,-D, MAX negative)	n.a.
P23	44	F	L	N	Y	PHEO	Sp, no screening	n.a.
P24	64	F	L	N	N	PHEO	Sp, no screening	n.a.
P25	19	M	L	N	N	PHEO	SDH-B	Pseudohypoxia
P28	43	F	L	N	Y	PHEO	RET	Kinase signaling
P29	66	F	L	N	Y	PHEO	Sp, no screening	n.a.
P30	42	F	Paraaortal L	N	N	PGL	Sp, (RET, VHL, SDH-B,-D negative)	n.a.
P31	66	M	R	N	Y	PHEO	Sp, no screening	n.a.
P32	25	M	GVR	N	n.a.	PGL	SDH-B	Pseudohypoxia
P33	68	F	L	N	Y	PHEO	Sp, no screening	n.a.
P34	63	M	L	N	Y	PHEO	Sp, (RET, VHL, SDH-B,-C,-D, MAX, TMEM127 negative)	n.a.
P35	50	F	Paraaortal L	N	Y	PGL	n.a.	n.a.
P36	64	F	R	N	Y	PHEO	Sp, no screening	n.a.
P37	54	M	R	N	N	PHEO	Sp, no screening	n.a.
P38	13	M	R	N	n.a.	PHEO	Sp, (RET, VHL, SDH-B,-D negative)	n.a.
P39	12	M	L	N	Y	PHEO	VHL	Pseudohypoxia
P40	47	M	L	N	N	PHEO	NF1	Kinase signaling
P41	42	F	L	N	Y	PHEO	Sp, no screening	n.a.
P42	51	F	Paraaortal	Y	Y	PGL	SDH-B	Pseudohypoxia
P44	33	M	R	N	Y	PHEO	RET	Kinase signaling
P45	66	F	L	N	Y	PHEO	Sp, no screening	n.a.
P46	72	F	L	N	n.a.	PHEO	Sp, no screening	n.a.
P47	13	M	Liver	N	n.a.	PGL	Sp, (RET, VHL, SDH-B,-D negative)	n.a.
P48	59	M	L	N	Y	PHEO	Sp, no screening	n.a.
P49	57	F	Cervical	Y	Y	PGL	SDH-B	Pseudohypoxia
P50	72	M	L	N	Y	PHEO	Sp, no screening	n.a.
P51	68	F	L	N	Y	PHEO	Sp, no screening	n.a.
P52	40	M	R	N	Y	PHEO	Sp, no screening	n.a.
P53	49	F	R	N	Y	PHEO	Sp, no screening	n.a.
P54	33	M	R	N	N	PHEO	Sp, (RET, VHL, SDH-B,-D negative)	n.a.
P55	29	F	L	N	N	PHEO	Sp, no screening	n.a.
P72	78	M	L	N	Y	PHEO	Sp, no screening	n.a.
P73	63	F	L	N	Y	PHEO	Sp, no screening	n.a.
P79	56	F	R	N	Y	PHEO	Sp, no screening	n.a.
P86	21	F	R	N	N	PHEO	RET	Kinase signaling
P88	56	F	R	N	Y	PHEO	Sp, no screening	n.a.
P89	18	F	R	N	n.a.	PHEO	VHL	Pseudohypoxia
P90	73	F	L	N	Y, Takotsubo	PHEO	Sp, no screening	n.a.
P107	55	F	L	N	n.a.	PHEO	Sp, no screening	n.a.
P118	22	M	n.a.	N	n.a.	PHEO	VHL	Pseudohypoxia
P119	50	M	R	N	N	PHEO	Sp, no screening	n.a.

Supplementary Table 2. Clinical details of the PHEO/PGL patients. Sp: sporadic cases, R: right, L: left, Abd: abdominal, GVR: glomus vagal right.

Supplementary Table 3

Gene	Protein encoded	Primer sequences (5'-3')
**TH**	Tyrosine Hydroxylase	**F:**GGC CGT GCA GCC CTA CCA AG<br>**R:**ACG GAG AAG GGG CGC TGG AT
**DDC**	Dopa Decarboxylase	**F:**TCT GCC CTG CAG GAA GCC CT<br>**R:**TTG TGG TCC CCA GGG TGG CA
**DBH**	Dopamine Beta-Hydroxylase	**F:**GGC CGG GAG TGG GAG ATC GT<br>**R:**TGT GGC CAG CTC CCG GTC TT
**PNMT**	Phenylethanolamine N-Methyltransferase	**F**:CGG GGG CCT TCA ACT GGA GC<br>**R**: GCACGTCGATGGGCAGGACC
**COMT**	Catecholamine-O- Methyltransferase	**F:**GGA ATG TGG CCT GCT GCG GA<br>**R:**CCG CGC ACG TGT GCT AGG AA
**MAOA**	Monoamine Oxydase A	**F:**GGG GGC TGC TAC ACG GCC TA<br>**R:**TCC TGC CCA CGG GTT GAC GA
**SLC18A1**	Vesicule Monoamine Transporter 1	**F:**TGG GTC GGT GGC TGT GTT CC<br>**R:**AGG CCA AGC CCT GCA TTG GG
**SLC18A2**	Vesicule Monoamine Transporter 2	**F:**CCA CGC TGC TGA AGG ACC CG<br>**R:** ACG CCC AGC TGC CAC TTT CG
**CHGA**	Chromogranin A	**F:** TCC CTG TGA ACA GCC CTA TG<br>**R:** AGG ATC CGT TCA TCT CCT CG
**CHGB**	Chromogranin B	**F:**ATG AAG GAA TGG TGA CTC G<br>**R:**TGT CTC TTT GTC TTT GAC GTC
**SYP**	Synaptophysin	**F:**GGA CAT GGA CGT GGT GAA TC<br>**R:**ATG TGG CAA AGG CGA AGA TG
**HPRT1**	Hypoxanthine Phosphoribosyl -transferase 1	**F:**TGA CAC TGG CAA AAC AAT GCA<br>**R:**GGT CCT TTT CAC CAG CAA GCT
**SDHA**	Succinate Dehydrogenase Complex Flavoprotein Subunit A	**F:**TGG GAA CAA GAG GGC ATC TG<br>**R:**CCA CCA CTG CAT CAA ATT CAT G
**TBP**	Tata-box binding protein	**F**:GCC CGA AAC GCC GAA TAT A<br>**R**:CGT GGC TCT CTT ATC CTC ATG A
**EEF1A1**	Eukaryotic Translation Elongation Factor 1 Alpha 1	**F**:CTG AAC CAT CCA GGC CAA AT<br>**R**:GCC GTG TGG CAA TCC AAT
**GAPDH**	Glyceraldehyde-3-Phosphate Dehydrogenase	**F**:CAT CCA TGA CAA CTT TGG TAT CGT<br>**R**:CCA TCA CGC CAC AGT TTC C

Supplementary Table 3: Primer sequences for qPCR. F: forward, R: reverse.
